# Development and psychometric testing of a scale assessing the sharing of medical information and interprofessional communication: the CSI scale

**DOI:** 10.1186/1472-6963-14-126

**Published:** 2014-03-13

**Authors:** Emmanuelle Anthoine, Christelle Delmas, Julie Coutherut, Leïla Moret

**Affiliations:** 1 PHU11, Public Health Department, University Hospital, Saint-Jacques Hospital, 85, rue Saint-Jacques, Nantes Cedex 44093, France; 2 EA 4275 SPHERE: bioStatistics, Pharmacoepidemiology and Human Sciences Research team, Faculty of Pharmaceutical Sciences, University of Nantes, Rue Gaston Veil, Nantes 44000, France; 3 Centre Fédératif Prévention Dépistage, University Hospital, Bâtiment Le Tourville, Nantes Cedex 44093, France

**Keywords:** Interprofessional communication, Patient information, Patient safety, Questionnaire, Psychometric properties, Scale validation

## Abstract

**Background:**

Interprofessional collaboration is essential in creating a safer patient environment. It includes the need to develop communication and coordination between professionals, implying a better sharing of medical information. Several questionnaires exist in the literature, but none of them have been developed in the French context. The objective was to develop and test the psychometric properties of the communication and sharing information (CSI) scale which assesses specifically interprofessional communication, especially the sharing of medical information and the effectiveness of communication between members of the team.

**Methods:**

The questionnaire construction process used a literature review and involved a panel of voluntary professionals. A list of 32 items explored the quality of shared information delivered to patients and the effectiveness of interprofessional communication. The study was conducted in 16 voluntary units in a University Hospital (France), which included medical, surgical, obstetrics, intensive care, pediatrics, oncology and rehabilitation care. The scale-development process comprised an exploratory principal component analysis, Cronbach’s α-coefficients and structural equation modeling (SEM).

**Results:**

From these 16 units, a total of 503 health professionals took part in the study. Among them, 23.9% were physicians (n = 120), 43.9% nurses (n = 221) and 32.2% nurse assistants (n = 162).

The validated questionnaire comprised 13 items and 3 dimensions relative to “the sharing of medical information” (5 items), “communication between physicians” (4 items) and “communication between nurses and nurse assistants” (4 items). The 3 dimensions accounted for 63.7% of the variance of the final questionnaire. Their respective Cronbach’s alpha coefficients were 0.80, 0.87 and 0.81. SEM confirmed the existence of the 3 latent dimensions but the best characteristics were obtained with a hierarchical model including the three latent factors and a global “communication between healthcare professionals” latent factor, bringing the 8 items linked to communication together. All the structural coefficients were highly significant (P < 0.001).

**Conclusions:**

This self-perception CSI scale assessing several facets of interprofessional communication is the first one developed in the French context. The development study exhibited excellent psychometric properties. Further psychometric analysis is needed to establish test-retest reliability, sensibility to change and concurrent validity.

## Background

In recent years, much attention has been paid to quality and safety of hospital care. Many authors have argued that this depends on an organization that ensures the continuity of information and interprofessional collaboration [1,2]. In fact, team working seems to play a major role in creating safer patient care [3,4]. The collaboration between physicians and nursing staff has been widely studied, in particular in intensive care units [5-7]. In intensive care units, it has been shown that effective collaboration between health care professionals will improve patient outcomes, as well as reduce medical errors, In addition, good cooperation between nurses and physicians is a characteristic of the “magnet hospitals”, which have lower nurse turnover and greater job satisfaction [5,8,9].

Although numerous studies have documented the benefit of effective team collaboration, especially between nurses and physicians, a collaborative model between physicians and nurses continues to be the exception [5,10]. The lack of collaboration and communication, and its consequent negative impact on the provision of healthcare and patient outcomes has been pointed out for years [11,12].

Several authors have defined and modeled this complex and multidimensional phenomenon of interprofessional collaboration [13-16]. Among existing definitions of this concept, Henneman [14] described it as “labouring together, sharing communication and decision-making …” as Baggs and Schmitt [13]. For these authors, collaboration means “nurse and physician cooperatively working together, sharing responsibility for solving problems and making decisions to formulate and carry out plans for patient care”. More recently, interprofessional collaboration has been defined as “the process of developing and maintaining a partnership between a team of health professionals and a client in a participatory, collaborative and coordinated approach to share decision-making around health and social issues” [17].

Most of the authors agree that the field of interprofessional collaboration includes the need to develop communication and coordination between professionals, and to better share medical information and problem-solving strategies. The Canadian Interprofessional Health Collaborative (CIHC) developed an integrative approach composed of six competency domains: interprofessional communication, patient-centred care, role clarification, team functioning, collaborative leadership and interprofessional conflict resolution [16]. The two first domains support the others. Communication skills seem to be essential for all professionals and involve the ability to communicate effectively with others, especially those from other professions. Furthermore a study concluded that “physicians are from Mars and nurses are from Venus”, noting that the lack of communication can be attributed to various reasons [18]. There are indeed many reasons, ranging from the nature of the nurse-physician relationship to differing work philosophies, responsibilities, social status or culture, gender inequality, and competence of nurses [18-20]. Casey underlines that physicians and nurses train separately, keep separate patient records, report to different hierarchies, read different journals, and use different jargon [21]. Moreover, a study carried out by our team showed that nurses do not know what the patient has been told, that this information is not recorded in the patient’s file, and that as a result it is difficult for them to adapt what they say to patients, which in turn has an impact on the quality and coherence of the information actually delivered [22]. However, information provided to patients has become a central part of care provision. Patients want to be involved in their own health care, and they cannot make the right decisions about treatment if they are not sufficiently informed about the possible side effects and complications, as well as how their treatment might affect their daily lives. In hospitals, multiple interactions exist, which in general involve several physicians as well as paramedical professionals, who may or may not be working in a structured team. This work organization, involving many different health professionals, not only requires good communication but also effective coordination of the different actions undertaken, in order to avoid repetition, inconsistency, or incoherence in caring for patients.

So, improvement programs for healthcare professional practices, focusing on communication and collaborative practices, are imperative to develop quality and safety of hospital care. To bring about change, professionals need to assess their levels of team collaboration, in particular their communications skills to share medical information so as to ensure coordination and continuity of care.

### Existing measures of interprofessional communication

Several questionnaires exploring interprofessional collaboration exist in the literature [15,23-26]. A review of 5 instruments validated in English was published by Dougherty and Larson in 2005 [27]. More recently, several other scales have been validated and published in different cultures [28-31]. Most of them only focused on physician-nurse collaboration, and explored different dimensions of performance or/and collaboration. Three of them contain a subscale specifically dedicated to interprofessional communication [24,28,30], but two of them were too recently published and therefore couldn’t be used. Only one recent scale was interested in sharing medical information [30]. None of the scales published before 2007 were able to answer specifically to our questioning. In addition none of them have been developed in the French language, except a scale exploring the multiple dimensions of organizational performance published in 2005 by Minvielle [24], which was only dedicated to intensive care units. Moreover, only a few have been psychometrically well validated. So the absence of such an instrument in the French context has made it challenging for hospitals to measure interprofessional communication.

The main objective of this paper is to develop and test the psychometric properties of the communication and sharing of information (CSI) scale which assesses a major facet of interprofessional collaboration in hospital settings, that is to say, interprofessional communication. Interprofessional communication includes the sharing of medical information and the effectiveness of communication between members of the team (physicians, nurses and nurse assistants), and is recognised as a major factor of interest in quality and safety management practices.

## Methods

### Instrument development (November 2008 – May 2009)

The first step was to identify instruments used to measure physician-nurse collaborative practices, especially those interested in communication and sharing information about patients and participating in decision-making concerning patient care. A literature search was carried out using Pubmed® using the following terms: nurse-physician, professionals, communication, collaboration, cooperation, sharing of information, questionnaire, scale, instrument and validation. When the study was conducted, only the papers published in English or French between 1990 and 2007 were examined. Several instruments have been developed and validated but none of them were specifically designed to answer our objective. Based on the literature review, a draft questionnaire compiled 33 items related to the dimensions of these instruments related to interprofessional communication, but none of them explored sharing of information. After the elimination of redundancies, we were left with 12 items.

The second step involved a panel of voluntary professionals (composed of 12 senior and junior physicians, 4 head nurses, 1 nurse and a director). The first meeting used the brainstorming methodology to generate ideas questioning good practices of sharing of medical information. All the ideas were then compiled from which 8 specific items were created. The second meeting was dedicated to a brainstorming concerning items generation related to interprofessional communication. The first list of 12 items was completed by the professionals’ views: they added 12 more items. These items were not new ideas but specific or completed interprofessional communication items by exploring relationships between identified professionals (for example, the original item from Minvielle [24] was divided into 2 items: “it’s easy to discuss patients with medical staff” and in “it’s easy to discuss patients with nursing staff”. This item was specified as follows: “it’s easy to discuss patients with medical staff”, “it’s easy to discuss patients with a head nurse”, “it’s easy to discuss patients with nurses”, “it’s easy to discuss patients with nurse assistants”).

Another meeting, which concerned the last part of the questionnaire, asked if tools for the sharing of information delivered to patients in units existed, and asked professionals about their proposals and needs for conducting improvement actions. Finally a meeting allowed to review the items of the questionnaire for clarity and content validity.

A list of 63 items in four parts was retained. The two first parts (32 items) explored the quality of information sharing delivered to patients and interprofessional communication. Response choices were a 4-point Likert-scale format. Items concerning characteristics of the sample were added: occupation, working hours, experience in the unit (number of years), experience in the hospital (number of years), age and sex. To ensure face validity (level of understanding, acceptability and time required to complete the questionnaire), the questionnaire was pilot tested by 5 health professionals (1 physician, 2 nurses and 2 nurse assistants). Three items were modified slightly in response to their comments.

**Figure 1:**
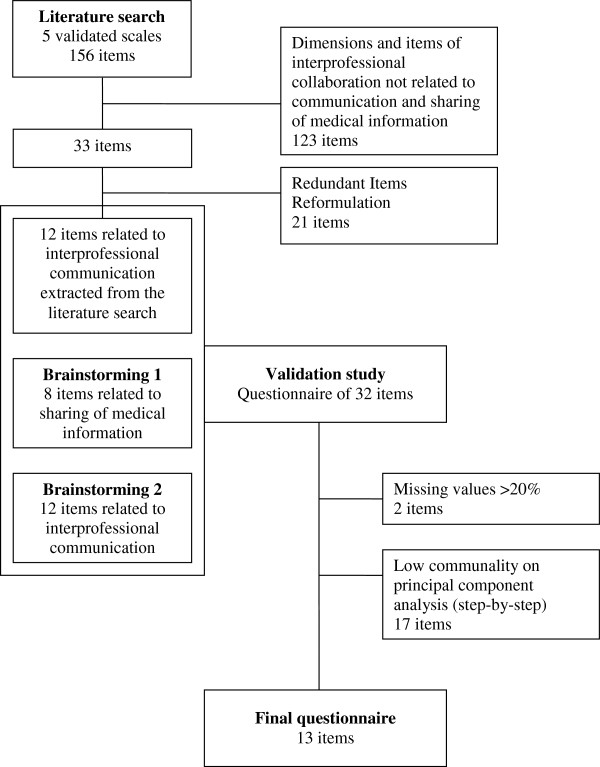
Qualitative and quantitative phases of scale development and validation (Flow chart).

The Flow chart provided in Figure 1 shows the qualitative and quantitative phases of the scale development and validation (Figure 1).

### Testing the questionnaire (June - July 2009)

The study was conducted in 16 voluntary units in medicine, surgery, obstetrics, intensive care units, pediatrics, oncology and rehabilitation care in the Nantes University Hospital (France).

### Scale-construction process (construct and convergent validity, reliability)

Statistical analyses used the usual techniques of descriptive statistics (frequency, means ± SD) and Pearson’s correlation coefficients between items two-by-two. Psychometric analyses were done in several steps: the first step consisted of eliminating items with a rate of missing values (missing value and “I don’t know” choice) >20%, or a floor or ceiling effect >50% [32]. The second step was an exploratory principal component analysis (PCA) using a varimax rotation for the remaining items [32]. The number of dimensions was determined using both a scree plot and clinical relevance of items. Two criteria were used to attribute each item to one of the dimensions: like other authors [33], factor loading >0.60 with one principal component was chosen, instead of factor loading >0.40 [34], and when an item exhibited factor loading across several dimensions, it was attributed to the one for which it maximized internal consistency assessed by Cronbach’s α-coefficient [34]. Finally, the homogeneity of the dimensions was assessed using convergent validity (correlation of each dimension item with all the other items in the dimension >0.40), divergent validity (correlation of each dimension item with all the other items in the other dimensions <0.40) and correlations between dimensions [35]. Cronbach’s α-coefficients were computed for evaluating internal consistency [34,36]. In order to ensure known group validity, a comparison of scores was done between units where a regular interprofessional meeting exists (i.e. a definite organization of interprofessional collaboration) and others that don’t have regular meetings. The final questionnaire was provided as an Additional file 1.

### Score calculation

Four response choices were offered, from never to always, with points attributed to each one (0, 1, 2 or 3), with higher values corresponding to better interprofessional communication. Individual scores for all professionals who responded to at least half of the items plus one in a dimension were calculated by additioning the responses to the items and then dividing that value by the number of items completed. The mean score for a dimension was the sum of individual scores divided by the number of respondents. Scores ranged from 0 to 100.

### Structural equations modelling (SEM) – partial least squares (PLS) approach

Structural equation modeling was performed to confirm factorial structure and unidimensionality of the dimensions. SEM is a comprehensive statistical approach to test hypotheses about relationships among observed and latent variables (dimensions) [37]. Parameter estimation used the PLS approach developed by Wold [38]. Results were read in two steps: evaluation of the quality of the measurement model in each dimension, with communality indexes which have to be superior or equal to 50% [39]; and then evaluation of the quality of the structural model, with redundancy indexes. Finally, the GoF index (Goodness of Fit) evaluated the quality of the global model. It had to be superior or equal to 0.9.

Characteristics of the sample were described by computing percentages, means, standard deviations and ranges. All study analyses were computed using R 2.15.1 and SPAD 5.6. SEM was performed using XLSTAT - PLSPM.

### Ethical and consent considerations

All physicians, nurses and nurse assistants were invited to participate by their head nurses. All professionals received a questionnaire accompanied by a letter in which the purpose of the study was explained. They were asked to return the questionnaire anonymously in a drop box on the ward. Participation was voluntary, filling in the questionnaire was considered as informed consent.

According to the articles L1121-1 and R1121-2 from the French code of public health, IRB approval was unnecessary.

## Results

### Characteristics of the sample

A total of 503 health professionals participated from 16 units of Nantes University Hospital. Among them 23.9% were physicians (n = 120), 43.9% nurses (n = 221) and 32.2% nurse assistants (n = 162). Participation rates were respectively 22%, 41% and 30% for a global rate of participation of 30%.

The majority of the sample were women (82.2%). Mean ± standard deviation (SD) age of the respondents was 37.2 ± 9.3 years and median was 36 years. Median [range] years of experience in the hospital was 8 years [0–40], and median [range] years of experience in the unit was 4 years [0–30].

### Scale-construction process

Among the 32 items, 2 were removed because their missing values rates were superior to 20%. They concerned the good communication between physicians when sharing information (22.8%) and the good communication between nurses and nurse assistants (27.9%).

No item had Pearson’s correlation coefficients with another item exceeding 0.6.

Five successive exploratory PCAs were then performed on the 30 remaining items and this led to the identification of three dimensions according to the scree plot. Seventeen items were removed step-by-step because of their low factor loadings on one of the three factors or because they exhibited factor loadings on both (Figure 1). No item maximized Cronbach’s α-coefficient and all the 13 remaining items had factor loading >0.60 within their own dimension.

The final questionnaire was a 13 items scale (Table 1). The first dimension was composed of five items exploring the sharing of medical information between healthcare professionals and accounted for 22.4% of the variance. The second one, which explored the effectiveness of communication between medical staff members, was composed of four items and accounted for 21.7% of the variance. The third one, which explored the effectiveness of communication between nurses and nurse assistants, was composed of four items and accounted for 19.6% of the variance. Their respective Cronbach’s α-coefficients were 0.80, 0.87 and 0.81. Correlations between items within a given dimension all exceeded 0.40 and correlations between one item and those of other dimensions were <0.40. The statistical parameters were reported in Table 2.

**Table 1:** Results of PCA using varimax rotation

		***Scale dimensions***		
***Short name***	***Label (in French and in English)***	***SH***^***1***^	***CM***^***2***^	***CNM***^***3***^
SH1	Les médecins et les infirmières partagent l’information qu’ils ont délivrée au patient ou reçue de celui-ci	**0.69**	0.25	0.01
	*Physicians and nurses share medical information received from or delivered to the patient*			
SH2	Les infirmières connaissent l’information médicale qui a été délivrée au patient	**0.79**	0.02	-0.07
	*Nurses know medical information that was delivered to patients*			
SH3	Les aides soignantes connaissent l’information médicale qui a été délivrée au patient	**0.77**	-0.02	-0.12
	*Nurse assistants know medical information that was delivered to patients*			
SH4	Il existe une discussion ouverte entre médecins, infirmières et aides soignantes autour de l’information médicale à délivrer au patient	**0.69**	0.31	0.07
	*Physicians, nurses and nurse assistants discuss medical information to be delivered to the patients*			
SH5	Les médecins et les infirmières collaborent pour décider de l’information médicale à délivrer au patient	**0.73**	0.06	0.01
	*Physicians and nurses collaborate to decide what medical information should be delivered to the patients*			
CM1	Il est facile de discuter des patients avec les médecins	0.04	**0.87**	-0.11
	*It’s easy to discuss patients with physicians*			
CM2	La communication est très facile entre les médecins du service	-0.11	**0.81**	-0.08
	*Communication is very easy between physicians.*			
CM3	La communication est très facile entre les autres soignants et les médecins du service	-0.21	**0.77**	0.19
	*Communication is very easy between physicians and other healthcare professionals*			
CM4	Il est facile de demander des conseils aux médecins du service	-0.13	**0.80**	-0.15
	*It’s easy to ask physicians for advice in the unit*			
CNM1	Il est facile de discuter des patients avec les infirmières	0.07	0.22	**0.73**
	*It’s easy to discuss patients with nurses*			
CNM2	Il est facile de discuter des patients avec les aides soignantes	0.01	0.04	**0.79**
	*It’s easy to discuss patients with nurse assistants*			
CNM3	Il est facile de demander des conseils aux infirmières du service	-0.08	0.17	**0.80**
	*It’s easy to ask nurses for advice in the unit*			
CNM4	Il est facile de demander des conseils aux aides soignantes du service	-0.12	0.06	**0.79**
	*It’s easy to ask nurse assistants for advice in the unit*			

^1^SH dimension: Medical information sharing between healthcare professionals.

^2^CM dimension: communication between physicians.

^3^CNM dimension: communication between nurses and nurse assistants.

Footnote: Factor loadings of the items in each dimension are highlighted in bold.

**Table 2:** Psychometric properties of the CSI scale

	***SH***	***CM***	***CNM***	***CM + CNM***	***Global***
**Item properties**					
# of items in the scale	5	4	4	8	13
% of questionnaires with at least ½ the items completed	99.6%	97.6%	98.1%	97.9%	98.1%
# of questionnaires with ½ + 1 items completed	530	510	513	523	524
# of items with “missing data” > 20%	0	0	0	0	0
# of items with “does not apply” response > 20%	0	0	0	0	0
# of item with ceiling effect > 50%	0	0	4	4	4
# of item with floor effect > 50%	0	0	0	0	0
**Scaling properties**					
Mean score (±SD)	49.3 (17.9)	69.3 (23.1)	86.9 (15.5)	78.1 (15.8)	-
Skewness value/SE	0.25/0.78	-0.59/0.78	-1.01/0.78	-0.38/0.78	-
Median	46.2	66.1	91.5	78.7	-
Ceiling effect (%)	0.9%	15.7%	47.7%	13.9%	-
Floor effect (%)	0%	1.2%	0%	0%	-
# of item correlation with own scale > 0.40	5	4	4	-	-
# of item correlation with own scale greater than with other scale	5	4	4	-	-
Cronbach’s α coefficient	0.80	0.87	0.81	0.83	-
Sum of square of the factors before rotation	31.6%	18.6%	13.5%	-	63.7%
% of variance explained by the factor	22.4%	21.7%	19.6%	-	63.7%
**Test of unidimensionality**					
1st eigenvalue	2.81	3.01	2.64	3.52	-
2nd eigenvalue	0.78	0.43	0.76	1.83	-
Dillon-Goldstein’s Rho	0.87	0.92	0.88	0.88	-

Moreover, in some units the sharing of medical information had already been developed thanks to regular interprofessional meetings. In order to ensure known group validity, a comparison of the “the sharing of medical information between healthcare professionals” score was done between those units and others. It was significantly higher (p < 0.01) in units having this specific organization (53.2 ± 10.5) than in others (45.7 ± 6.1).

Based on scores ranging from 0 to 100, mean (± SD) scores were 49.3 ± 17.9 for “the sharing of medical information between healthcare professionals” dimension, 69.3 ± 23.1 for “the effectiveness of communication between medical staff members” dimension and 86.9 ± 15.5 for “the effectiveness of communication between nurses and nurse assistants” dimension. Results are reported in Table 2.

### Structural equations modelling (SEM)

**Figure 2:**
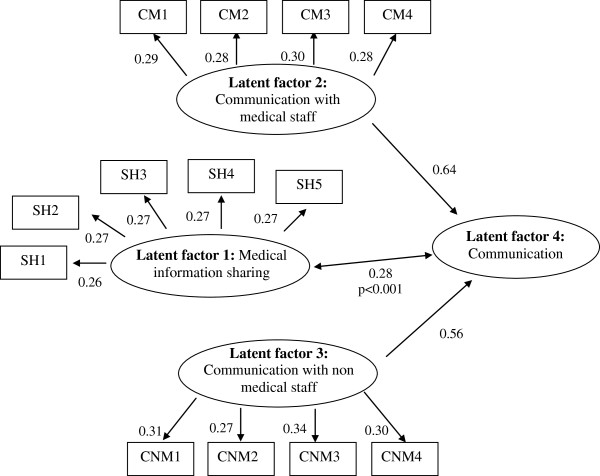
Structural Equations Modelling – PLS approach.

SEM confirmed the existence of 3 latent dimensions (“1-The sharing of medical information between healthcare professionals”, “2-The effectiveness of communication between medical staff members” and “3-The effectiveness of communication between nurses and nurse assistants”), but the best characteristics were obtained with a hierarchical model including the three latent factors and a global “The communication between healthcare professionals” latent factor, bringing 2 dimensions together (Figure 2)

Goodness of fit of the data to the model was very good: communality indexes of the three dimensions were 0.56, 0.66 and 0.75 respectively. The redundancy index was 0.49 and the global GOF index was 0.95. All the structural coefficients were highly significant (P < 0.001).

## Discussion

Our study produced the validated CSI scale consisting of 13 items and 3 subscales measuring several elements of interprofessional communication, including the sharing of medical information between healthcare professionals, communication between physicians and communication between nurses and nurse assistants. Confirmatory analysis (SEM) supported the possibility of calculating a global score of communication between healthcare professionals bringing together the two dimensions related to interprofessionals’ communication. This self-perception questionnaire is the first one developed in the French context. It exhibited excellent psychometric properties. The results of this study provide evidence for high construct, divergent and discriminant validity. Internal consistency was over 0.80 as recommended [34,36]. Items had strong factor loadings with the three principal component analysis-identified factors and accounted for more than 2/3 of the variance. For modelling, the PLS approach was chosen because of the non-normality of the data [38]. The statistical validation strategy presented herein follows most of the recommendations of 'good practice’ for score validation [40].

This scale is short and easy to use. It explores healthcare professionals’ perception of their ability to communicate and to share medical information delivered to patients. These dimensions are key factors in patient satisfaction and safety [1-3]. The instrument is usable by the whole team, including nurse assistants and is not limited to intensive care units.

Nevertheless, there are a number of study limitations concerning the methodology of the study. First, the study sample was chosen by convenience and may not be representative of the variety of settings and of the general population of healthcare workers. Although our study included several different kinds of units, it was not exhaustive. Selection bias may be present because healthcare professionals who have a particular interest in promoting interprofessional communication are perhaps more likely to have responded to the questionnaire. However, while the rate of participation by healthcare professionals was amply sufficient to obtain relevant data for the validation study, it cannot be seen as a reliable estimate of the perception that professionals have in the Nantes university hospital, and so our results cannot be generalised. As this was the first time that nurse assistants were interviewed on this large sample, it may explain why they, in particular, were reluctant to respond. Secondly the date at which the survey was conducted (June and July), close to the holiday period, could also be a factor that reduced the response rate. The reasons why some professionals did not answer the questionnaire remain to be explored. The sample is issued from a big university hospital in one region of France only, and results might be different in other hospitals and regions. The application of the findings to all hospitals is therefore limited.

A number of limitations concerning the instrument development can also be noted. First, the expert review of the items, from outside experts, did not occur to validate the items in the initial instrument. Secondly, our instrument only explored two facets of the concept of interprofessional communication, that is to say the sharing of information and communication between team members. Moreover interprofessional communication is measured within a multi-disciplinary practice perspective, and not within a collaborative practice perspective. Thirdly, we hypothesized that health professionals understand and can articulate what constitute interprofessional communication. Finally, Table 2 showed that the dimension related to communication between nurses and nurse assistants had an important ceiling effect and that the distribution of the score was not normal. A further validation process is required to reduce this ceiling effect, for example a test of a new response pattern. Further psychometric analysis is also needed to establish test-retest reliability, sensitivity to change, concurrent validity and cross-cultural validations in other countries. Moreover, our items might also be validated by comparing them against the descriptors for the CIHC’s interprofessional competency domain [16], and our findings should be completed by more work considering others dimensions of interprofessional collaboration assessment. According to authors, developed instruments propose specific dimensions about coordination [28,29], cooperation [29], partnership [29], problem solving, decision making process [29,30], and leadership [25,41].

## Conclusion

The CSI scale exploring interprofessional communication should assist researchers and quality managers who wish to assess levels of effective communication in clinical units. It should help team members to explore how they work and share information together in order to enhance their healthcare practices. The sharing of medical information within the healthcare team ensures the quality of information delivered to the patient. Conversely, absence of cohesion and inconsistencies between physicians and nursing staff, and their different modes of expression towards the patient, often generate anxiety and result in insufficient information reaching the patient. One line of approach is therefore the improvement of communication between physicians and nurses/nurse assistants. They could both benefit from a better definition of their roles, by undertaking further training in conflict-resolving, in effective methods of asserting their own opinions and knowledge, in listening skills, and in conducting collaborative ward rounds. These methods for training teams, in order to improve interpersonal skills, have already been validated in the field of aviation or other industries, and in hospitals they have also shown their value in improving the quality and safety of care delivered to the patient.

## Competing interests

The authors declare that they have no competing interests.

## Authors’ contributions

EA realized the statistical analysis, interpretation of results, and took part in writing the manuscript. CD made a review of the literature and has been involved in drafting the manuscript. JC has been involved in the design of the study and in the data collection. LM conceived the study, coordinated the study and took part in writing the manuscript. All authors have read and approved the final manuscript.

## Pre-publication history

The pre-publication history for this paper can be accessed here:

http://www.biomedcentral.com/1472-6963/14/126/prepub

## Supplementary Material

Additional file 1 Communication and sharing of information Scale (CSI scale). Click here for file
