# Supplementary material for: Development and psychometric testing of a scale assessing the sharing of medical information and interprofessional communication: the CSI scale
Source: BMC Health Serv Res. 2014 Mar 13;14:126. doi: 10.1186/1472-6963-14-126 (PMC4008265; doi:10.1186/1472-6963-14-126)
Supplement: Additional file 1 — Communication and sharing of information Scale (CSI scale). [file 1472-6963-14-126-S1.doc]

**Communication and sharing of information Scale (CSI scale)**

We would like to know your opinion about the way you communicate and share of medical information between members of the staff. Your answers are completely confidential, so please be as open and honest as you can. Thank you very much for your help.

**Please use this scale to give your opinion. Check the box corresponding to your answer for each item.**

|  | Never | Sometimes | Often | Always |
| --- | --- | --- | --- | --- |
| **Medical information sharing between healthcare professionals** | | | | |
| 1- Physicians and nurses share medical information received from or delivered to the patient | ❑ | ❑ | ❑ | ❑ |
| 2- Nurses know medical information that was delivered to patients | ❑ | ❑ | ❑ | ❑ |
| 3- Nurse assistants know medical information that was delivered to patients | ❑ | ❑ | ❑ | ❑ |
| 4- Physicians, nurses and nurse assistants discuss medical information to be delivered to the patients | ❑ | ❑ | ❑ | ❑ |
| 5- Physicians and nurses collaborate to decide what medical information should be delivered to the patients | ❑ | ❑ | ❑ | ❑ |
| **Communication between medical staff members** | | | | |
| 6- It’s easy to discuss patients with physicians | ❑ | ❑ | ❑ | ❑ |
| 7- Communication is very easy between physians | ❑ | ❑ | ❑ | ❑ |
| 8- Communication is very easy between physicians and other healthcare professionals | ❑ | ❑ | ❑ | ❑ |
| 9- It’s easy to ask physicians for advice in the unit | ❑ | ❑ | ❑ | ❑ |
| **Communication between nurses and nurse assistants** | | | | |
| 10- It’s easy to discuss patients with nurses | ❑ | ❑ | ❑ | ❑ |
| 11- It’s easy to discuss patients with nurse assistants | ❑ | ❑ | ❑ | ❑ |
| 12- It’s easy to ask nurses for advice in the unit | ❑ | ❑ | ❑ | ❑ |
| 13- It’s easy to ask nurse assistants for advice in the unit | ❑ | ❑ | ❑ | ❑ |
